# Unleashing potential: evaluating the effectiveness of the BOPPPS teaching strategy in Chinese urology education

**DOI:** 10.3389/fmed.2025.1452165

**Published:** 2025-03-05

**Authors:** Rong-ling Zhang, Kai Gan, Hong Zuo, Donghui Han, Kang Shi, Jing Wang, Keying Zhang, Wenkai Jiang, Diya Wang, Yu Li

**Affiliations:** ^1^College of Educational Technology, Northwest Normal University, Lanzhou, Gansu, China; ^2^School of Education, Lanzhou City of University, Lanzhou, Gansu, China; ^3^Department of Urology, Xijing Hospital, The Fourth Military Medical University, Xi'an, Shaanxi, China; ^4^Department of Dialysis, No.88 Hospital, Taian, Shandong, China; ^5^Academic Affairs Office, The Fourth Military Medical University, Xi'an, Shaanxi, China; ^6^Medical Affairs Office, Third Affiliated Hospital, The Fourth Military Medical University, Xi'an, Shaanxi, China; ^7^State Key Laboratory of Oral & Maxillofacial Reconstruction and Regeneration & National Clinical Research Center for Oral Diseases & Shaanxi Key Laboratory of Stomatology & Department of Operative Dentistry and Endodontics, School of Stomatology, The Fourth Military Medical University, Xi'an, Shaanxi, China; ^8^Department of Occupational and Environmental Health and the Ministry of Education Key Lab of Hazard Assessment and Control in Special Operational Environment, School of Public Health, The Fourth Military Medical University, Xi'an, China; ^9^Department of Urology, Institute of Surgery Research, Daping Hospital, Army Medical University, Chongqing, China

**Keywords:** BOPPPS, urology, education, meta analysis, visual reality (VR), traditional lecture-based learning (LBL)

## Abstract

**Introduction:**

The BOPPPS teaching strategy has gained popularity in medical education in China as a more effective and practical pedagogy. However, its impact on knowledge acquisition and clinical skills in urology education has not been comprehensively evaluated. This study seeks to assess the effectiveness of the BOPPPS strategy in comparison to traditional lecture-based learning (LBL) during clinical internships in Chinese urology education, utilizing meta-analysis for verification.

**Methods:**

A cohort of 96 clinical medicine students from Xijing Hospital, engaged in clinical practice at the Department of Urology from September 2022 to June 2023, were stratified into two groups and exposed to identical teaching materials. The experimental group (n=48) was instructed using the BOPPPS model, while the control group (n=48) adhered to traditional instructional methods. Data on student satisfaction and self-assessment of the course were collected through a questionnaire, and end-of-course performance was evaluated through a post-study examination. We used meta-analysis aimed to evaluate the overall effectiveness of the BOPPPS teaching strategy compared to LBL teaching in surgery-related medical education.

**Results:**

The experimental group, which received instruction using the BOPPPS teaching model, achieved significantly higher scores in theoretical knowledge assessments and clinical practical skills compared to the control group. Additionally, the experimental group demonstrated greater levels of interaction with both teachers and students, with instructors displaying a higher ability to foster independent thinking among students. Furthermore, the teaching process in the experimental group was found to utilize classroom time more efficiently in comparison to the control group. And we confirmed that the BOPPPS model demonstrated a greater capacity to stimulate student interest in urology and improve their overall proficiency by meta-analysis.

**Discussion:**

The BOPPPS model exhibits superior efficacy in clinical teaching of urology, thus warranting consideration for wider adoption and dissemination.

## Introduction

Urology is a crucial surgical specialty that places a strong emphasis on clinical reasoning and the resolution of complex clinical challenges (1). Hence, the stringent demands for doctors’ fundamental theoretical understanding and practical skills are emphasized (2). Within the conventional urological educational framework in China, clinical instructors are heavily occupied with patient care, thereby relegating trainee doctors to primarily observational roles (3). The level of interaction between clinical educators and medical trainees is inadequate and ineffective (4). Furthermore, due to the sensitive nature of urological diseases and the importance of maintaining patient privacy, trainee doctors frequently encounter limitations when observing treatments or conducting urological examinations (5). Minimally invasive technology is integral to the advancement and modernization of urology, as well as in practical instruction (6). However, due to constraints in medical teaching resources and operating environments, interns are often unable to gain hands-on experience with advanced technologies like laparoscopy and the Da Vinci surgical robot, thus limiting their acquisition of relevant practical skills (7, 8). In summary, novice medical practitioners have limited chances to acquire practical experience, resulting in a tenuous connection between theoretical understanding and application in clinical settings.

Traditional lecture-based learning (LBL) instruction serves as the primary method for imparting fundamental knowledge to medical students during their clinical rotations, with an emphasis on the instructor and the delivery of syllabus content and concepts (9). The internship experience may be perceived as tedious by trainee doctors, leading to a lack of motivation for engaging in active learning (10). This lack of motivation hinders the development of essential clinical skills, critical thinking abilities, and effective doctor-patient communication. Therefore, it is imperative to reform the conventional teaching approach in China to foster trainee doctors’ problem-solving skills and cultivate a new generation of highly skilled medical professionals.

In contemporary medical education, traditional teaching methods often adhere to the BOPPPS instructional framework, which stands for Bridge-in, Objectives and Outcomes of Learning, Pre-assessment, Participatory learning, Post-assessment, and Summary. It was first introduced by Douglas Kerr from the University of British Columbia (11), and has been developed recently in medical education in China. According to the constructivist learning theory, the BOPPPS teaching strategy provides a comprehensive framework and process for attaining instructional goals (12). The BOPPPS model prioritizes comprehensive engagement and reciprocal communication between educators and medical trainees, with a central emphasis on the trainees themselves.

Over the last decade, the BOPPPS teaching strategy has been implemented in the instruction of various medical subjects, across a wide range of medical disciplines, including but not limited to dental materials education (11), ophthalmology education (13), oral histopathology education (14), physiology education (15) and gynecology education (16). There is currently a lack of literature regarding the implementation of the BOPPPS model in urology education. While the BOPPPS model has demonstrated success and effectiveness in enhancing students’ academic knowledge, its applicability in urology education for clinical medical students in China remains uncertain. In this study, a cohort of 96 undergraduate students who have completed clinical probation in the urology department of Xijing Hospital were selected to investigate the varying impacts of integrating the BOPPPS model with Virtual Reality (VR) technology and conventional Lecture-Based Learning (LBL) approaches in urology education. And we also use meta-analysis aimed to evaluate the overall effectiveness of the BOPPPS teaching strategy compared to LBL teaching in surgery-related medical education.

## Methods

### Participants

This observational study was conducted among final-year undergraduate medical trainee doctors at the Department of Urology, Xijing Hospital, from September 2022 to June 2023. All participants provided informed consent for their involvement in the study. A control group of 48 interns utilized the traditional Lecture-Based Learning (LBL) teaching method, while an experimental group of 48 interns utilized the BOPPPS model combined with Virtual Reality (VR) technology. The control group comprised 7 females and 41 males, while the experimental group included 9 females and 39 males. Both cohorts of students utilized the urology related diseases from a common textbook as instructional material. The teaching procedures were conducted concurrently in both the experimental and control groups. Patients were chosen from the inpatient population of the urology department for use as a teaching case. Prior to the instructional session, the teacher engaged in communication with the patients and secured their consent. Subsequently, the teacher compiled the pertinent medical information of the patient into a case study. The specific pedagogical approaches employed are outlined.

### Traditional LBL model

The control group was mainly taught by the traditional LBL teaching method. The teacher first explained the relevant theoretical knowledge of the selected disease according to the syllabus’s specific requirements. Afterwards, students discussed and answered clinical questions based on the cases provided by the teacher. Finally, the teacher summarized the course content according to the requirements of the syllabus.

### BOPPPS model

One week prior to the commencement of the internship, the instructor provided the students with an overview of the theoretical chapters and associated topics. The BOPPPS model was delineated into six distinct stages.

***Bridge-in***: Based on the instructional material, prior to the internship, an online teaching platform was established and teaching resources were uploaded, encompassing three typical representative cases of urological diseases, pertinent theoretical knowledge, recent literature, as well as videos and images of clinical procedures, thereby enhancing the study’s focus and practical significance from basic to advanced levels.

***Learning objective***: According to the syllabus, the teacher emphasized the specific requirements and key points of theoretical knowledge and clinical skills for urological diseases.

***Pre-assessment***: Following the establishment of learning objectives, medical trainees were allotted a day for independent study. Subsequently, they participated in an online theoretical examination and interactive interview designed to assess their proficiency in case analysis and theoretical knowledge prior to commencing their internship. The instructional administrator then evaluated the examination outcomes, pinpointing areas of high error frequency for clinical instructors to prioritize during the internship period.

***Participatory learning***: Initially, the trainee doctors were organized into groups and tasked with selecting classic cases that aligned with the instructional content of the discussion. Subsequently, a spokesperson from each group was designated to address the questions pertaining to the selected cases. Ultimately, the instructor provided feedback on the responses from each group and elucidated key and challenging aspects of the cases. Students were encouraged to refer to textbooks and scholarly literature, share gathered information, engage in group deliberations, analyze and synthesize the posed questions, and collaborate to enhance their understanding.

To enhance trainee doctors’ theoretical understanding and practical skills, the instructor utilized the resources of a clinical skills training center to develop a 3D model of urological anatomy using VR simulation and 3D body software. This model was employed to elucidate the physiological structure and pathological morphology of the urinary system in detail. In the context of practical implementation of minimally invasive surgery, novice medical practitioners can acquire proficiency in utilizing minimally invasive surgical instruments by engaging with virtual reality simulators and da Vinci surgical robot operating systems. Additionally, they can enhance their skills in instrument manipulation through exercises in instrument handling and suturing, thereby fostering proactive learning and improving overall efficiency.

***Post-assessment***: The post-assessment, conducted on the final day of the rotation, comprised an online theoretical examination and an offline clinical skills assessment. The instructors emphasized the complex theoretical concepts related to diseases of urology in formulating the post-assessment queries. By utilizing the post-assessment to gage students’ comprehension of the instructional material, educators can refine the difficulty level of the curriculum and enhance its efficacy.

**Figure 1:**
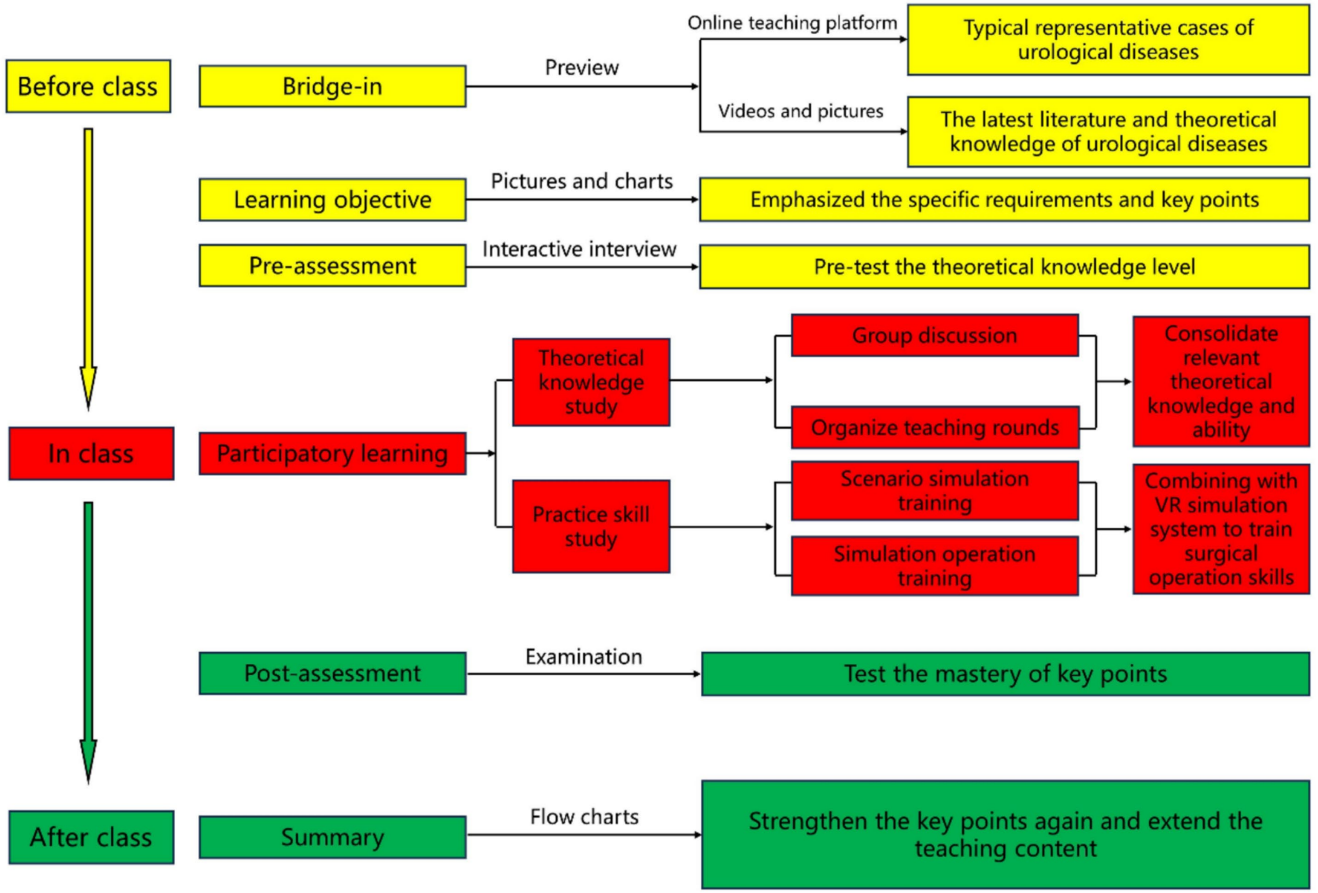
Flow chart of class design for BOPPPS model.

***Summary***: The educators utilized a flow chart to assist students in synthesizing the lecture material, reinforcing key concepts, addressing challenging points, and expanding the scope of instruction. Additionally, clinical instructors responded to inquiries posed by medical trainees and elaborated on the lesson content by referencing the chapters on urological diseases within the course. The BOPPPS model flowchart is depicted in Figure 1.

### Effectiveness assessment

This study primarily assessed the effectiveness of integrating the BOPPPS teaching method with virtual reality technology in enhancing the mastery of professional theoretical knowledge, clinical thinking skills, practical abilities, and overall satisfaction among urology interns. All participants underwent standardized evaluations on theoretical knowledge and clinical skills, with the former being assessed through a closed-book examination comprising multiple-choice, definition, and case analysis questions. The clinical skills assessment comprises evaluations of clinical practice operations and clinical comprehensive abilities. Both student cohorts are administered identical test papers, with uniform marking criteria and scores assigned on a 100-point scale for each component.

Moreover, the effectiveness and satisfaction of the course were assessed through a questionnaire survey. A total of 96 questionnaires were distributed, all of which were successfully returned, resulting in a 100% recovery rate. According to the teaching evaluation guidelines outlined by the Cornell Center (11), the questionnaire primarily encompasses assessments of instructors’ professional knowledge, clarity of instructional content, level of student-teacher interaction, responsiveness to student inquiries, enhancement of independent thinking skills, promotion of active student engagement in practical activities, cultivation of interest in the subject matter, and evaluation of the efficient utilization of class time. Each survey response was rated on a 5-point scale, with scores ranging from 1 to 5.

### Meta-analysis

This meta-analysis and systematic review were designed according to the PRISMA (preferred reporting items for systematic reviews and meta-analyses, PRISMA) guidelines (17). The PICOS (population, intervention, comparison, outcome, and study design) framework was used to determine the inclusion criteria of studies. The following studies will be included: (a) the participants for the studies were medical students in Chinese medical schools; (b) the experimental group received the intervention of BOPPPS teaching strategy; (c) the groups of LBL were as control; (d) the core curriculums covered clinical medicine and/or biomedicine disciplines; (e) the studies were two-group controlled (randomized/nonrandomized); (f) the outcomes presented as data or descriptions of each controlled studies included at least one of the following measurements: PSS, KES, TS; (g) only studies fulltext published in English language and Chinese language were included. (h) All mentioned studies conducted before 22 Nov 2023. Any study which did not meet the inclusion criteria was excluded. The key search terms included BOPPPS, medicine and student. PubMed and Chinese electronic databases of CNKI were searched before 22 Nov 2023. According to a predefined form, data were searched, collected, and extracted by two independent reviewers (R. Rong and K. Gan). The Cochrane risk of bias 2 (RoB2 v9) tool was used to evaluate the quality of individual included studies (18).

### Statistical analysis

Statistical analyses were conducted utilizing SPSS version 27.0 software and Microsoft Office. Measurement data were presented as mean ± standard deviation (x ± s) and significance was determined through an independent sample t-test with a threshold of *p* < 0.05.

## Results

### Comparison of general information between the two groups

The study sample consisted of 96 five-year undergraduate interns specializing in clinical medicine at the First Affiliated Hospital of Air Force Medical University, comprising 80 males and 16 females. Utilizing the random number table method, the experimental group was stratified into two groups: the experimental group receiving instruction through a combination of VR technology and the BOPPPS teaching model, and the control group receiving instruction through the traditional LBL model. As indicated in Table 1, each group consisted of 48 students. Analysis revealed no statistically significant disparities in demographic variables such as gender, age, and prior academic performance between the two groups (*p* > 0.05), thus ensuring comparability in the study population.

**Table 1:** Comparison of general information between the two groups.

Variable		Experimental group (*n* = 48)	Control group (*n* = 48)	*t*/*χ^2^* value	*p* value
Gender (%)	Male	39 (81.25)	41 (85.42)	0.3000	0.5839
	Female	9 (18.75)	7 (14.58)		
Age(year)		21.94 ± 0.9087	21.85 ± 0.9223	0.4459	0.6567
Prior academic performance(score)		84.42 ± 3.4450	84.64 ± 4.0680	0.0542	0.9569

### Comparison of the theoretical knowledge assessment of the two groups

**Figure 2:**
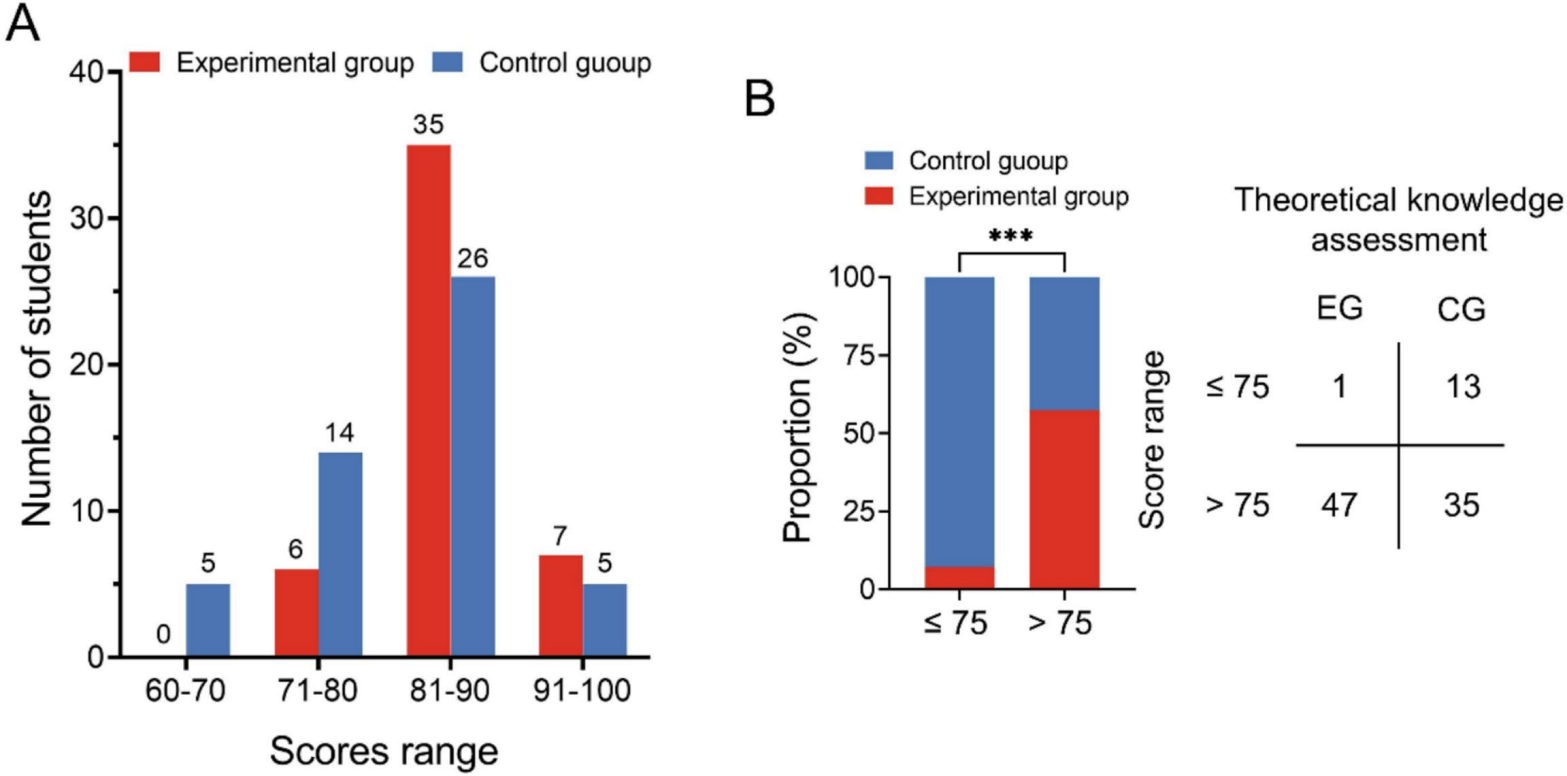
Comparison of the theoretical knowledge assessment of the two groups. **(A)** Distribution chart of students’ theoretical knowledge assessment scores; **(B)** The distribution of good grades and passing grades in the two groups (EG, Experimental group; CG, Control group; ***p < 0.001).

The results presented in Table 2 indicate that students in the VR technology combined with BOPPPS teaching group achieved a mean theoretical knowledge assessment score of (85.23 ± 4.673) points, while students in the traditional LBL model group scored (80.81 ± 7.151) points. The scores of the experimental group were found to be significantly higher than those of the control group, with a statistically significant difference (*p* < 0.05). Further analysis in Figure 2A reveals that the distribution of scores among students in the experimental and control groups. None of the students in the experimental group scored within the 60–70 points range. In the 71–80 points range, there were 6 students in the experimental group and 14 in the control group. For scores between 81 and 90 points, there were 35 students in the experimental group and 26 in the control group. In the 91–100 points range, there were 7 students in the experimental group and 5 in the control group. A score of 75 was used as the threshold between good grades and passing grades for further analysis of the theoretical assessment results of both groups. Figure 2B demonstrates a significantly higher number of students with good grades in the experimental group than that in the control group.

**Table 2:** Comparison of the theoretical knowledge assessment scores of the two groups.

Variable	Experimental group	Control group	*t* value	*p* value
Theoretical knowledge assessment (score)	85.23 ± 4.673	80.81 ± 7.151	3.582	< 0.05
Pass rate %	100	100	–	–

### Comparison of the clinical practical skills assessment of the two groups

**Figure 3:**
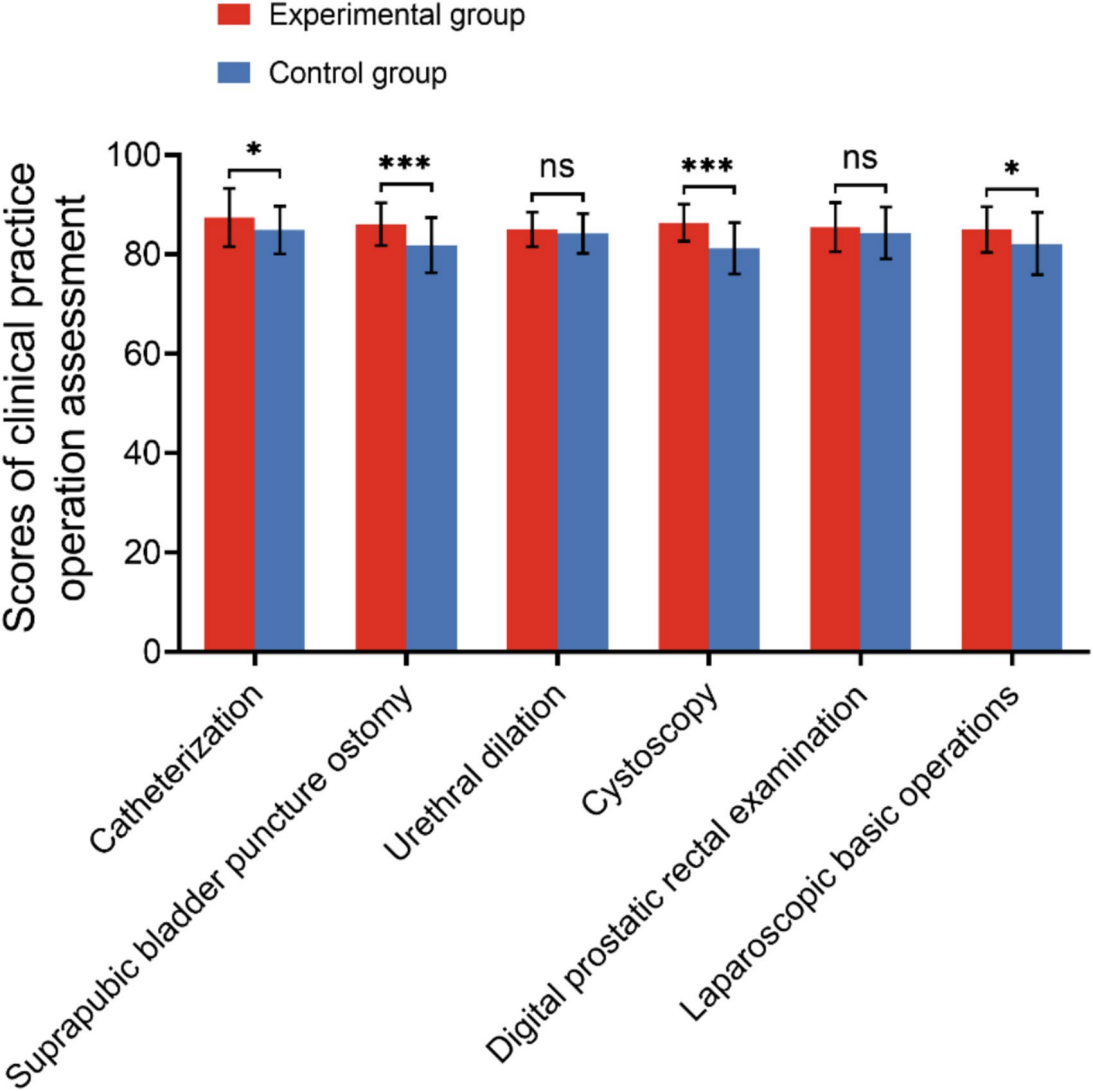
Scores of the clinical practice operation assessment of the two groups (ns, no significance; *p < 0.05; ***p < 0.001).

**Figure 4:**
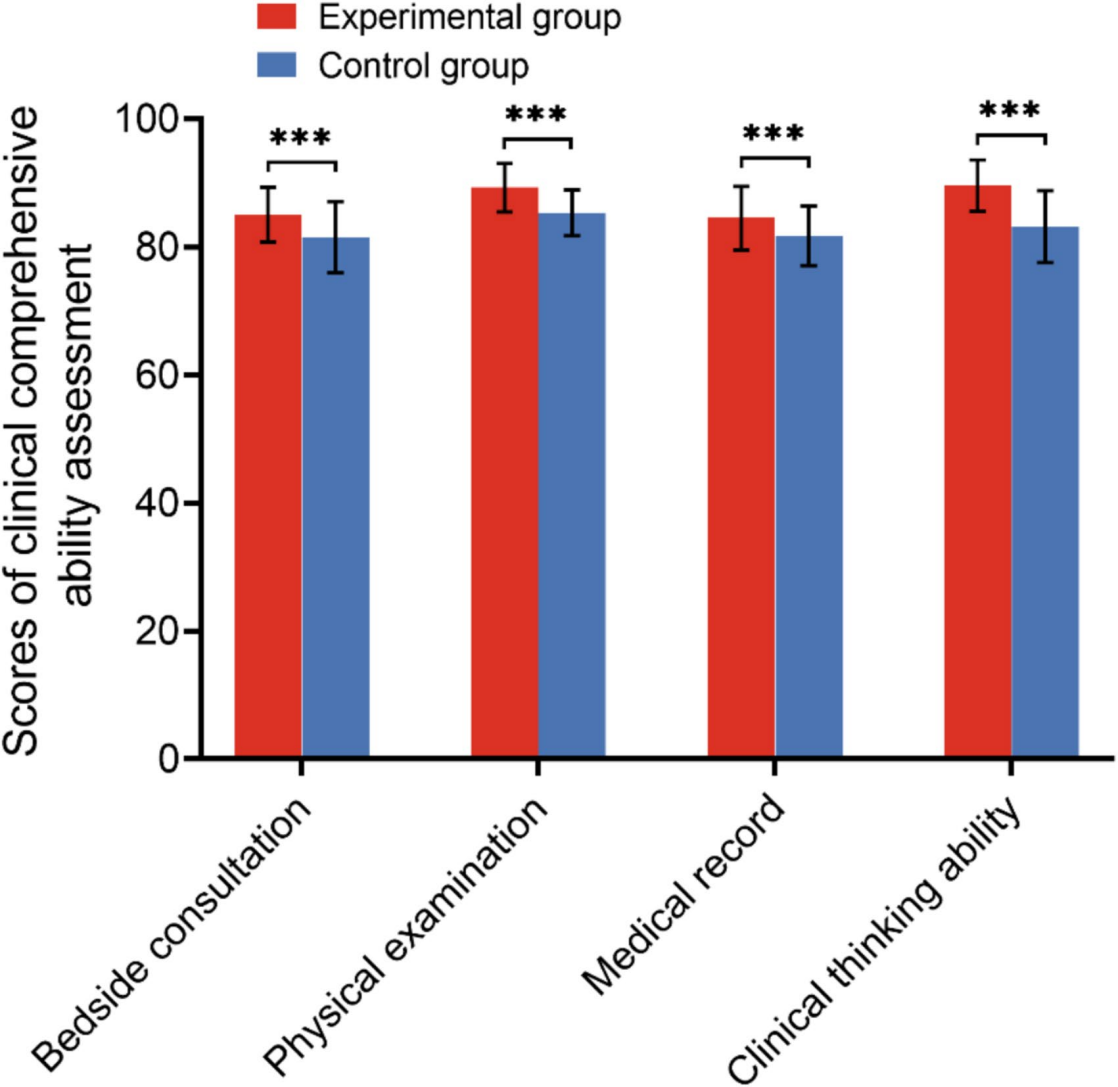
Scores of the clinical comprehensive ability assessment of the two groups (***p < 0.001).

The clinical practical skills assessment encompasses both the evaluation of clinical practice operations and the assessment of clinical comprehensive abilities. The clinical practice operation assessment includes procedures such as catheterization, suprapubic bladder puncture ostomy, urethral dilation, cystoscopy, digital prostatic rectal examination, and laparoscopic basic operations, totaling six items. The clinical comprehensive ability assessment evaluates skills in bedside consultation, physical examination, medical record writing, doctor-patient communication and clinical critical thinking abilities, totaling four items. During the assessment of clinical practice operations, the experimental group demonstrated statistically significant higher average scores in urinary catheterization, suprapubic bladder puncture ostomy, cystoscopy, and laparoscopic basic operations compared to the control group (*p* < 0.05). Conversely, there was no significant difference in the average scores between the two groups in urethral dilation and digital prostate-rectal examination, as illustrated in Figure 3 and Table 3. In the clinical comprehensive ability assessment, the average scores of the four assessment contents in the experimental group were significantly higher than those in the control group, as indicated by statistical analysis (*p* < 0.05) presented in Figure 4 and Table 4.

**Table 3:** Comparison of the clinical practice operation assessment of the two groups.

Variable	Catheterization	Suprapubic bladder puncture ostomy	Urethral dilation	Cystoscopy	Digital prostatic rectal examination	Laparoscopic basic operations
Experimental group	87.38 ± 5.859	86.06 ± 4.290	85.02 ± 3.492	86.35 ± 3.716	85.45 ± 4.959	84.98 ± 4.592
Control group	84.88 ± 4.832	81.83 ± 5.552	84.17 ± 4.028	81.21 ± 5.182	84.31 ± 5.219	82.15 ± 6.284
*t* value	2.282	4.176	1.110	5.591	1.103	2.522
*p* value	0.0247	< 0.01	0.2698	< 0.01	0.2730	0.0133

**Table 4:** Comparison of the clinical comprehensive ability assessment of the two groups.

Variable	Bedside consultation	Physical examination	Medical record	Clinical thinking ability
Experimental group	85.06 ± 4.260	89.27 ± 3.780	84.52 ± 4.968	89.60 ± 4.020
Control group	81.56 ± 5.558	85.38 ± 3.606	81.73 ± 4.653	83.19 ± 5.633
*t* value	3.463	5.167	2.842	6.424
*p* value	< 0.01	< 0.01	< 0.01	< 0.01

### Comparison of course effectiveness and satisfaction between the two groups

**Figure 5:**
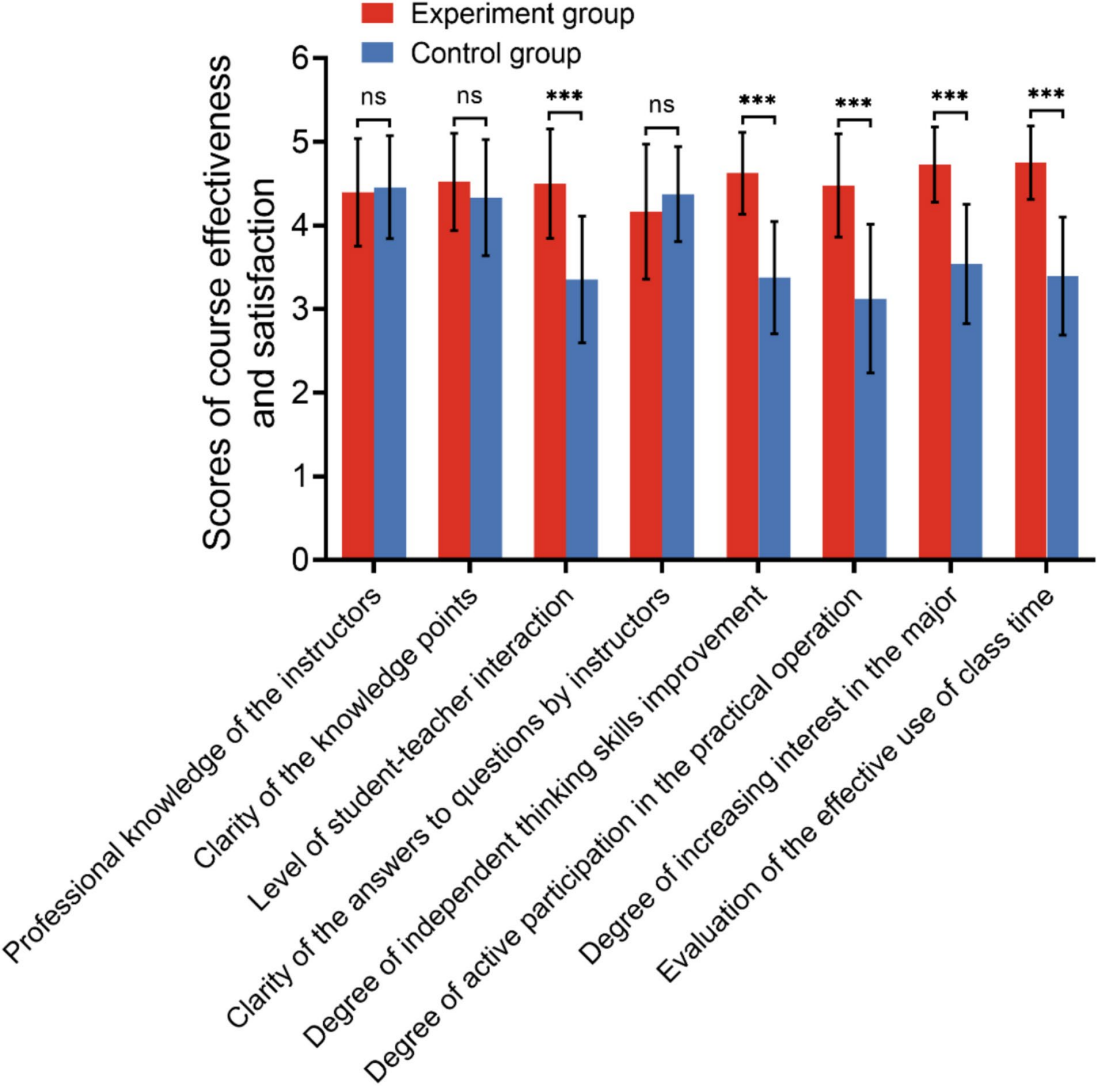
Scores of course effectiveness and satisfaction between the two groups (ns, no significance; *p < 0.05; ***p < 0.001).

The VR technology combined with the BOPPPS teaching model group exhibited a higher level of interaction with both teachers and students (*p* < 0.01), with instructors demonstrating a greater ability to promote independent thinking among students (*p* < 0.01). Furthermore, the teaching process in this group effectively utilized classroom time more efficiently (*p* < 0.01) compared to the control group. There was no statistically significant difference in the questionnaire results between the two groups regarding teachers’ professional knowledge reserves, clarity of knowledge points, and clarity of question answers. The findings indicate that the BOPPPS combined with VR technology teaching mode has the potential to enhance students’ critical thinking and active learning skills, as well as increase their satisfaction and recognition levels compared to traditional classroom teaching methods, as illustrated in Figure 5 and Table 5.

**Table 5:** Comparison of course effectiveness and satisfaction between the two groups.

Questionnaire items	Experimental group	Control group	*t* value	*p* value
Professional knowledge of the instructors	4.396 ± 0.6438	4.458 ± 0.6174	0.4854	0.6285
Clarity of the knowledge points	4.251 ± 0.5831	4.333 ± 0.6945	1.433	0.1553
Level of student-teacher interaction	4.500 ± 0.6523	3.354 ± 0.7576	7.940	< 0.01
Clarity of the answers to questions by instructors	4.167 ± 0.8078	4.375 ± 0.5696	1.460	0.1475
Degree of independent thinking skills improvement	4.625 ± 0.4892	3.375 ± 0.6724	10.41	< 0.01
Degree of active participation in the practical operation	4.479 ± 0.6185	3.125 ± 0.8903	8.655	< 0.01
Degree of increasing interest in the major	4.729 ± 0.4491	3.542 ± 0.7133	9.760	< 0.01
Evaluation of the effective use of class time	4.750 ± 0.4376	3.396 ± 0.7068	11.29	<0.01

The findings presented above are derived from our single-center investigation. However, recognizing the inherent limitations of single-center studies, such as methodological constraints and the restricted sample size, we conducted a systematic review and meta-analysis to enhance the robustness and objectivity of our conclusions. To achieve this, we aggregated data from multiple peer-reviewed studies, performed comprehensive statistical integration using validated analytical software, and synthesized the evidence to yield the following key results (Figures 4, 5).

### Effectiveness of BOPPPS strategy in medical education of surgery-related clinical internships: a systematic review and meta-analysis

#### Database searching and selection

**Figure 6:**
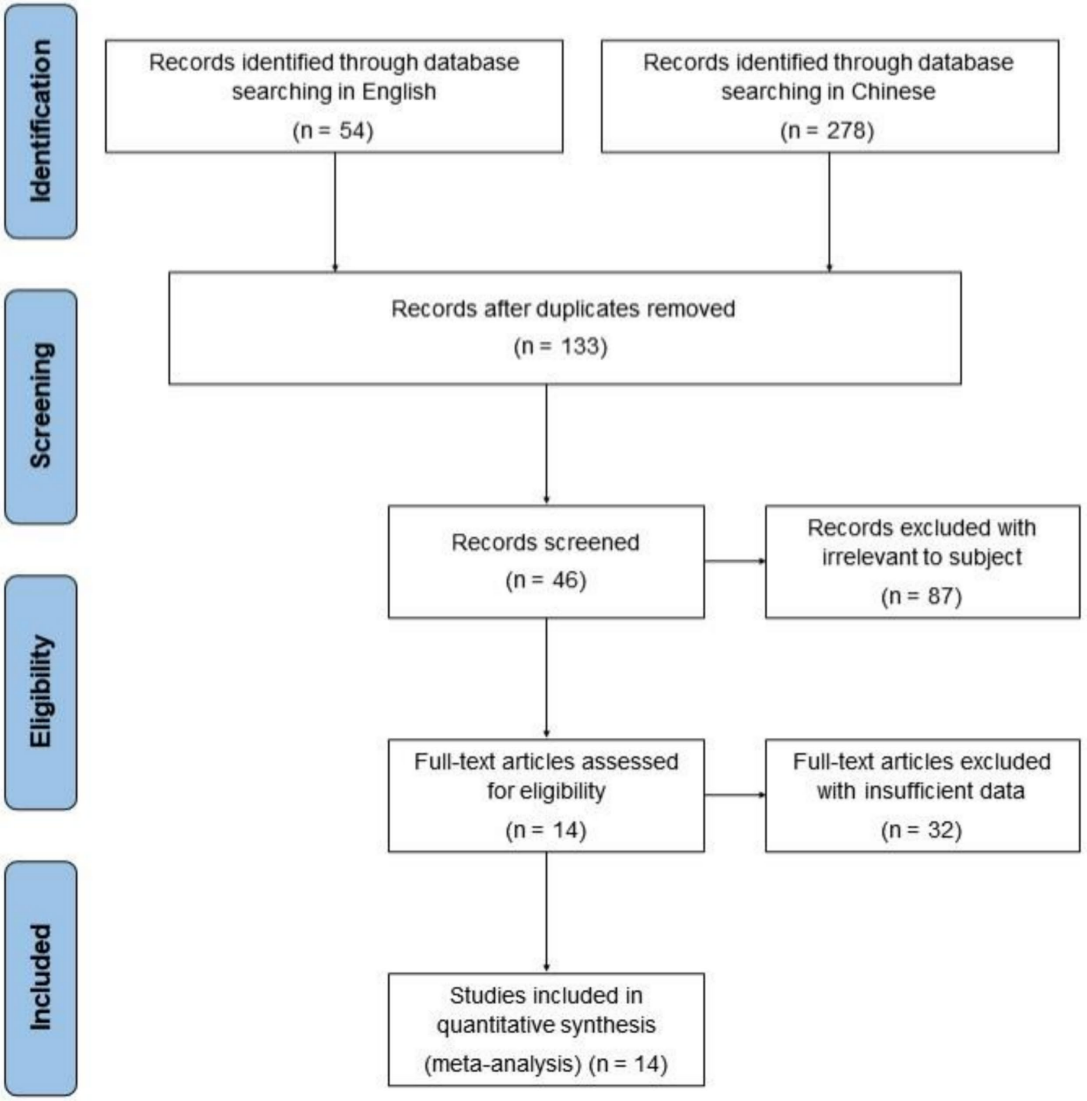
The methodological flowchart of PRISMA of the included studies in this meta-analysis.

The methodological flowchart of the Preferred Reporting Items for Systematic Reviews and Meta-Analyses (PRISMA) was presented in Figure 6. Initially, 332 potentially relevant records were retrieved from the electronic database, with 278 duplicate records being subsequently excluded. Following a review of the titles and abstracts, 87 publications were further excluded due to their lack of relevance to the subject of the meta-analysis, including those related to internal medicine, pharmacy, experience summaries, or questionnaire surveys without quantitative score measurements. After a thorough review of the complete text, an additional 32 articles were excluded due to insufficient data for extraction (*n* = 24) and/or lack of control trials (*n* = 8). Ultimately, 14 randomized controlled trials (RCTs) met the inclusion criteria and were included in the meta-analysis.

#### Study characteristics

The essential characteristics of the 14 selected studies are outlined in Table 6. The publication dates of these studies were prior to November 22, 2023. A total of 730 medical students participated in the BOPPPS teaching strategy, while 709 medical students were involved in the LBL approach across the 14 studies. All participants in the included studies were enrolled in medical school. Furthermore, all of the studies selected were randomized controlled trials (RCTs). Four trials focused solely on theoretical courses, one trial focused solely on practical courses, and nine trials examined both theoretical and practical courses.

**Table 6:** Main characteristics of the included studies in the current meta-analysis.

References	Study design	Sample size (BOPPPS)	Sample size (LBL)	Population	Course name	Course type	Outcome measures	RoB2
Bai et al. (36)	RCT	57	58	Undergraduates	Pediatric surgery	Theory and Practice	KES, PSS, TS	L
Chen et al. (13)	RCT	44	43	Undergraduates	Ophthalmology	Theory	KES, TS	L
Duan et al. (37)	RCT	55	52	Undergraduates	Orthopedics	Theory and Practice	KES, SS	L
Gu et al. (38)	RCT	30	30	Undergraduates	Neurosurgery	Theory and Practice	KES, PSS, TS	L
Hu et al. (39)	RCT	40	40	Undergraduates	Thoracic surgery	Theory	KES, TS	L
Hu et al. (29)	RCT	44	44	Undergraduates	Thoracic surgery	Theory	KES, TS	L
Jia et al. (40)	RCT	64	64	Undergraduates	Thoracic surgery	Practice	PSS, TS	L
Li et al. (41)	RCT	108	109	Undergraduates	Gynecology and obstetrics	Theory and Practice	KES, TS	L
Li et al. (30)	RCT	36	27	Undergraduates	Surgical nursing	Theory	KES, TS	L
Tao et al. (42)	RCT	52	52	Undergraduates	Surgery	Theory and Practice	KES, PSS, TS	L
Wang et al. (43)	RCT	25	25	Undergraduates	General surgery	Theory and Practice	KES, PSS, TS	L
Xu et al. (16)	RCT	121	114	Undergraduates	Gynecology	Theory and Practice	KES, PSS, TS	L
Yang et al. (11)	RCT	54	51	Undergraduates	Dental Materials	Theory and Practice	KES, PSS, TS	L
Zhang et al. (44)	RCT	50	50	Undergraduates	Neurosurgical nursing	Theory and Practice	KES, PSS, TS	L

RCT, randomized controlled trial; Undergraduates, medical students from freshmen to five-grade in the school; KES, knowledge examination score; PSS, practice skill score; TS, teaching satisfaction. RoB2, The Cochrane risk of bias 2; L, overall bias of low.

#### Evaluation of the effectiveness of BOPPPS teaching model compared with LBL model

##### Measurements of final knowledge examination scores

**Figure 7:**
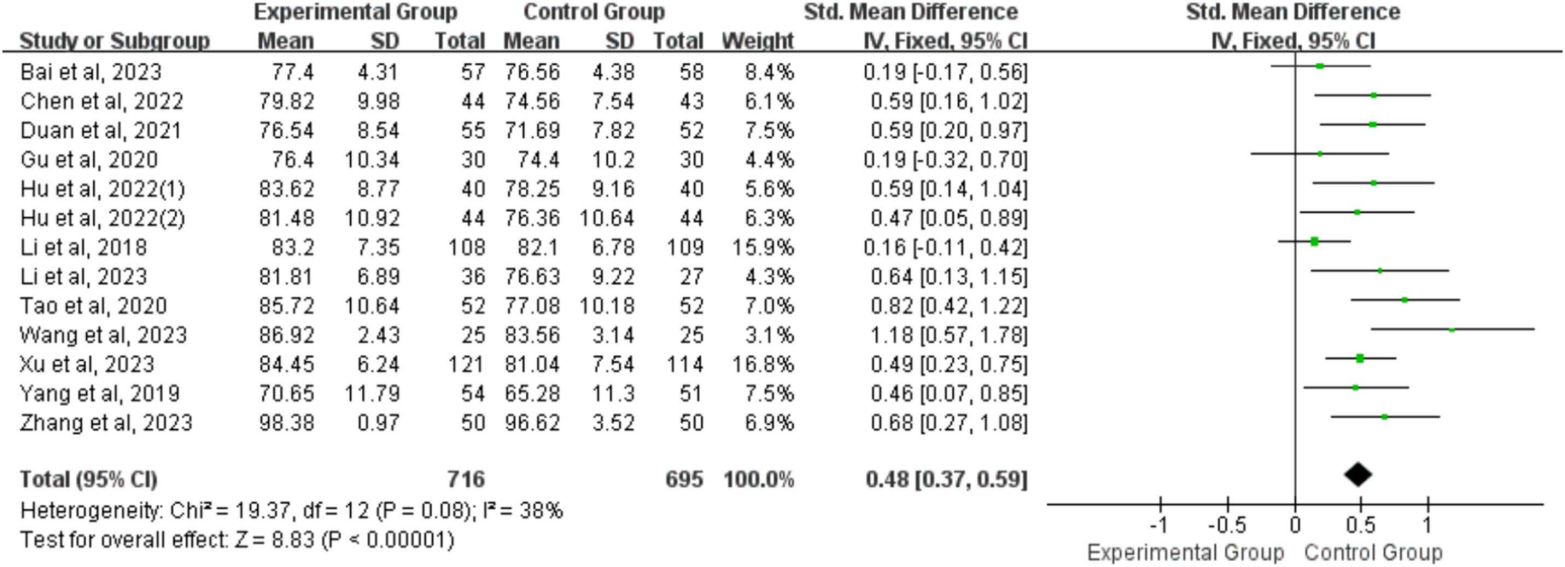
Forest plot of theoretical knowledge examination scores for BOPPPS teaching model compared with LBL model.

Thirteen studies were included in the final KES evaluation, encompassing a total of 716 and 695 students in the BOPPPS and LBL groups, respectively. The pooled effect size of these studies (SMD 0.48, 95% CI: 0.37–0.59, Z = 8.83, *p* < 0.00001) indicated a significant improvement in theoretical knowledge scores with a large effect size in the BOPPPS teaching strategy compared to LBL teaching. A fixed-effects model was employed for the meta-analysis due to the moderate heterogeneity (*p* = 0.08, I^2^ = 38% < 50%) observed in the data (Figure 7).

##### Measurements of practice skill scores

**Figure 8:**
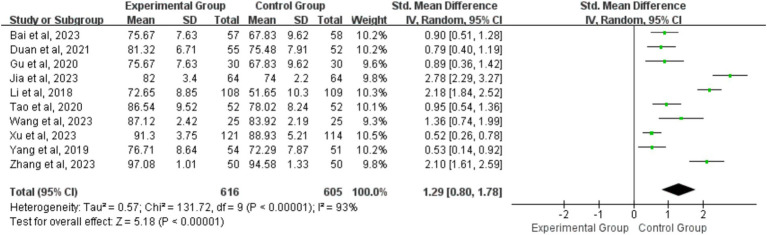
Forest plot of practice skill scores for BOPPPS teaching model compared with LBL model.

The analysis included data from 10 studies involving a total of 616 and 605 students in the BOPPPS and LBL groups, respectively, in relation to SS evaluation. In comparison to LBL teaching, the pooled effect of the 10 studies (SMD 1.29, 95% CI: 0.80–1.78, Z = 5.18, *p* < 0.00001) demonstrated a significant enhancement in SS within the BOPPPS group. The utilization of a random-effects model for the meta-analysis was warranted due to the notable statistical heterogeneity (*p* < 0.00001, I^2^ = 93%) observed among studies (Figure 8).

#### Quality assessment

**Figure 9:**
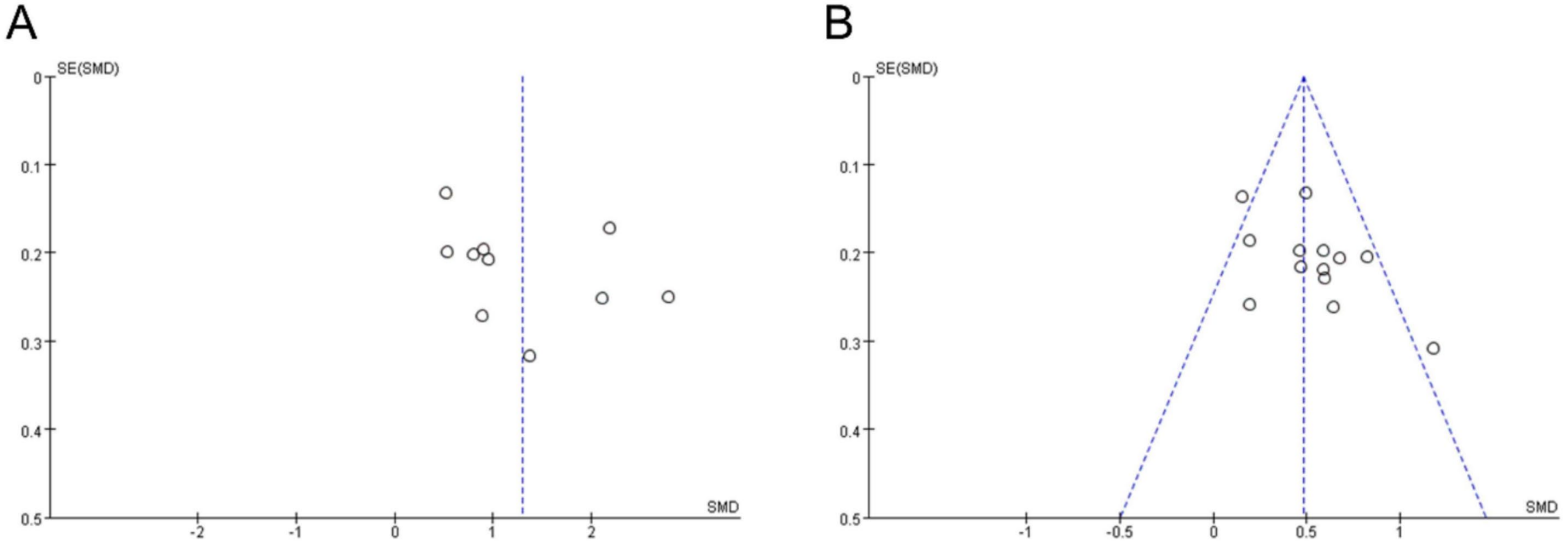
Funnel plots for publication bias. **(A)** Publication bias of practice skills scores; **(B)** Publication bias of knowledge examination scores.

Following the guidelines outlined in the Cochrane Collaboration Handbook, the assessment for each outcome included evaluation based on five domains: Selection of the reported result, Measurement of the outcome, Missing outcome data, Deviations from intended interventions, and Randomization process. Based on these domain ratings, the overall bias of each included study was determined to be at a “low risk of bias.” Subsequently, the funnel plot depicting the relationship between knowledge examination scores and practice skills scores exhibited near symmetry, suggesting minimal indication of substantial publication bias (Figure 9).

## Discussion

The projected rise in urological disease cases among an aging population will likely lead to an increased demand for medical providers in this field (19, 20). However, the current trend in medical school curricula shows a decreasing emphasis on urology education (1, 21). The primary obstacle facing medical schools in China is the training of competent clinicians who can effectively navigate the evolving hospital setting and address the healthcare needs of the Chinese population (22, 23). As educators in urology, it is incumbent upon us to optimize the learning outcomes for medical students during our limited yet influential interactions. In the conventional urological education framework in China, clinical instructors are frequently overburdened and often ill-equipped for internships, relying predominantly on didactic teaching methods (24). Consequently, medical trainees primarily serve as passive observers with limited hands-on experience, resulting in inadequate and inefficient interactions between instructors and trainees. Students often experience disengagement, lack of motivation, and suboptimal learning outcomes (24, 25). Hence, there is a pressing need for enhancing the quality of teaching, leading to the implementation of a teaching position filled by senior urologist attending physicians who dedicate 1 year to serving as a full-time teaching administrator. However, addressing the challenges of stimulating student interest in urology, enhancing clinical teaching effectiveness, and optimizing teaching methodologies are critical issues that require resolution in the current educational process.

The BOPPPS teaching strategy was first proposed by Douglas Kerrin from the University of British Columbia in 1978 (26). In contrast to LBL, the BOPPPS teaching strategy is not commonly utilized in medical education in numerous countries (27). This strategy offers a structured six-phase framework for developing learning activities, making it a valuable tool for educators seeking to deconstruct and evaluate their teaching methods in order to enhance student learning outcomes in medical education (28). The implementation of the BOPPPS teaching strategy in Chinese universities was first explored in 2011 with the aim of enhancing teaching efficacy and comprehensive skills in non-medical disciplines such as botany and English instruction (26). The BOPPPS model has recently been implemented and rigorously tested within China’s higher medical education sector with the aim of enhancing educational and learning efficacy through a structured teaching approach and fostering active student engagement (12). The main courses included are thoracic surgery (29), gynecology (16), surgical nursing (30), dental Materials education (11), physiology (15) and ophthalmology (13). This student-centered teaching method and observation system offers distinct advantages over conventional teaching methodologies by effectively stimulating student interest and enthusiasm for learning, thereby enhancing teaching efficiency. Moreover, the intricate nature of the urinary system presents a significant challenge in surgical education, with trainee physicians often struggling to comprehend its three-dimensional structure accurately (31–33). When compared to the conventional LBL model, the integration of VR technology with the BOPPPS model proves to be a more effective method for enhancing trainee understanding and proficiency (34, 35).

In this study, an analysis of urology theoretical knowledge assessment scores revealed a significant difference between the control group and the group exposed to a combination of VR technology and the BOPPPS teaching model. Despite both groups achieving a 100% pass rate, it is evident that students in the VR technology combined BOPPPS model group exhibit a superior grasp of complex concepts. Specifically, students in the experimental group achieved notably higher scores. Examination of Figure 2 further illustrates this disparity, with a greater number of students in the control group scoring between 60–70 and 71–80 compared to those in the experimental group. Conversely, students in the experimental group outperformed their counterparts in the control group when scores fell within the 81–90 and 91–100 range. Our study provides additional evidence to support the assertion that the experimental group exhibited a higher proportion of students with good grades compared to the control group. This suggests that the integration of VR technology with the BOPPPS model may facilitate the advancement of students from passing grades to higher levels of academic achievement. This finding aligns with the conclusions drawn by Hu et al. in their research on thoracic surgery education (29). The findings of the meta-analysis indicated a statistically significant increase in final knowledge examination scores among students in the BOPPPS group compared to those in the LBL group. This suggests that the BOPPPS teaching strategy has the potential to enhance students’ skills, intrinsic motivation in learning, and self-directed learning abilities, ultimately improving academic performance. Given the practical nature of urology, which places a strong emphasis on clinical reasoning and complex problem-solving skills, it is imperative for physicians to attain proficiency in clinical skills. In this study, the clinical skills assessment was segmented into the clinical practice operation assessment and the clinical comprehensive ability assessment. The findings indicated that, within the clinical skills assessment, the average scores of the VR technology combined with the BOPPPS teaching model group were significantly higher than those of the LBL model group in the more intricate subjects, such as urinary catheterization, suprapubic bladder puncture ostomy, cystoscopy, and laparoscopic basic operation. In the context of relatively straightforward topics such as urethral dilation and digital prostate-rectal examination, there was no statistically significant variance in the mean scores of the two cohorts. This finding suggests that the integration of virtual reality technology with the BOPPPS instructional approach may enhance students’ acquisition and proficiency in technical skills required for practical courses. In the context of a clinical comprehensive ability assessment, the mean scores of the group utilizing virtual reality technology in conjunction with the BOPPPS teaching model consistently surpassed those of the group employing the LBL model across the domains of bedside consultation, physical examination, medical record analysis, and clinical reasoning. The meta-analysis revealed that, relative to the LBL group, the BOPPPS group exhibited significantly elevated scores in practical skills, suggesting that the BOPPPS teaching strategy has the potential to enhance the motivation and engagement of medical students.

An additional significant metric for assessing the benefits of integrating VR technology with the BOPPPS teaching model is the efficacy of the course and student satisfaction. To mitigate the potential physical and mental strain associated with traditional “cramming” pedagogy, it is imperative to cultivate student engagement from the outset, as student satisfaction serves as a proxy for course effectiveness. The findings of this research indicate that participants in the experimental cohort expressed high levels of satisfaction with the instructional approach employed in the course. In contrast to conventional theoretical teaching methods, the BOPPPS model imposes elevated demands on educators, necessitating a departure from the traditional teacher-centered instructional approach. The involvement of students in the learning process poses challenges for educators, necessitating a high level of theoretical knowledge and extensive clinical practice experience. Upon further examination of the findings of this study, it was observed that students perceive the integration of VR technology with the BOPPPS teaching model as more effective in optimizing classroom time, increasing engagement with course material, and enhancing participation in hands-on activities compared to the traditional LBL approach. These results align with previous research in the field (26).

Nevertheless, this study was subject to various limitations. Firstly, the systematic literature search was limited to the databases of PubMed and CNKI, with criteria for inclusion and exclusion that may have been inadequate, suggesting a need to broaden the scope to include additional databases. Secondly, the absence of established guidelines for the implementation of BOPPPS in medical fields, as well as standardized criteria for evaluating the effectiveness of the BOPPPS teaching strategy in China, further constrained the study. Furthermore, this study utilized questionnaire surveys as an additional measurement to evaluate the efficacy of the BOPPPS teaching model, potentially introducing subjective bias. The study specifically focused on Chinese medical students and compared the impact of the BOPPPS teaching strategy versus LBL alone. Future research should aim to assess and contrast the effectiveness of BOPPPS in comparison to other teaching methodologies through Bayesian network meta-analysis.

In conclusion, the BOPPPS model is recommended as an open instructional design framework. Educators are encouraged to incorporate their extensive teaching expertise into their daily instructional routines while adhering to the principles of the BOPPPS teaching model. It is important to tailor the instructional design to the specific content being taught and the students’ existing knowledge base in order to align with their psychological characteristics and cognitive processes.

## Data Availability

The original contributions presented in the study are included in the article/supplementary material, further inquiries can be directed to the corresponding authors.

## References

[1] Casilla-Lennon M Motamedinia P. Urology in undergraduate medical education. Curr Urol Rep. (2019) 20:69. doi: 10.1007/s11934-019-0937-x, PMID: 31606783

[2] Mac Lennan S Duncan E Skolarus TA Roobol MJ Kasivisvanathan V Gallagher K. Improving guideline adherence in urology. Eur Urol Focus. (2022) 8:1545–52. doi: 10.1016/j.euf.2021.10.007, PMID: 34702647

[3] Cheng JW Wagner H Hernandez BC Ruckle HC. Consistencies and discrepancies between the expectations of urology trainees and the experience of practicing urologists. Urology. (2019) 127:42–8. doi: 10.1016/j.urology.2018.12.047, PMID: 30742865

[4] Matloubieh JE Eghbali M Abraham N. Strategies to encourage medical student interest in urology. Curr Urol Rep. (2020) 21:34. doi: 10.1007/s11934-020-00984-1, PMID: 32767185 PMC7411268

[5] Lebastchi AH Khouri RK Jr McLaren ID Faerber GJ Kraft KH Hafez KS. The urology applicant: an analysis of contemporary urology residency candidates. Urology. (2018) 115:51–8. doi: 10.1016/j.urology.2017.10.065, PMID: 29408686

[6] Eswara JR Ko DS. Minimally invasive techniques in urology. Surg Oncol Clin N Am. (2019) 28:327–32. doi: 10.1016/j.soc.2018.11.012, PMID: 30851832

[7] Mikhail D Sarcona J Mekhail M Richstone L. Urologic robotic surgery. Surg Clin North Am. (2020) 100:361–78. doi: 10.1016/j.suc.2019.12.003, PMID: 32169184

[8] Gostlow H Marlow N Babidge W Maddern G. Systematic review of voluntary participation in simulation-based laparoscopic skills training: motivators and barriers for surgical trainee attendance. J Surg Educ. (2017) 74:306–18. doi: 10.1016/j.jsurg.2016.10.007, PMID: 27836238

[9] Horne A Rosdahl J. Teaching clinical ophthalmology: medical student feedback on team case-based versus lecture format. J Surg Educ. (2017) 74:329–32. doi: 10.1016/j.jsurg.2016.08.009, PMID: 27651053

[10] Neyra JA Tio MC Ferrè S. International medical graduates in nephrology: a guide for trainees and programs. Adv Chronic Kidney Dis. (2020) 27:297–304.e1. doi: 10.1053/j.ackd.2020.05.003, PMID: 33131642

[11] Yang Y You J Wu J Hu C Shao L. The effect of microteaching combined with the BOPPPS model on dental materials education for Predoctoral dental students. J Dent Educ. (2019) 83:567–74. doi: 10.21815/JDE.019.068, PMID: 30858273

[12] Ma X Ma X Li L Luo X Zhang H Liu Y. Effect of blended learning with BOPPPS model on Chinese student outcomes and perceptions in an introduction course of health services management. Adv Physiol Educ. (2021) 45:409–17. doi: 10.1152/advan.00180.2020, PMID: 34018832

[13] Chen L Tang XJ Chen XK Ke N Liu Q. Effect of the BOPPPS model combined with case-based learning versus lecture-based learning on ophthalmology education for five-year paediatric undergraduates in Southwest China. BMC Med Educ. (2022) 22:437. doi: 10.1186/s12909-022-03514-4, PMID: 35668389 PMC9170341

[14] Wang S Xu X Li F Fan H Zhao E Bai J. Effects of modified BOPPPS-based SPOC and flipped class on 5th-year undergraduate oral histopathology learning in China during COVID-19. BMC Med Educ. (2021) 21:540. doi: 10.1186/s12909-021-02980-6, PMID: 34702232 PMC8546376

[15] Liu XY Lu C Zhu H Wang X Jia S Zhang Y. Assessment of the effectiveness of BOPPPS-based hybrid teaching model in physiology education. BMC Med Educ. (2022) 22:217. doi: 10.1186/s12909-022-03269-y, PMID: 35354465 PMC8966603

[16] Xu Z Che X Yang X Wang X. Application of the hybrid BOPPPS teaching model in clinical internships in gynecology. BMC Med Educ. (2023) 23:465. doi: 10.1186/s12909-023-04455-2, PMID: 37349730 PMC10286474

[17] Liberati A Altman DG Tetzlaff J Mulrow C Gøtzsche PC Ioannidis JP. The PRISMA statement for reporting systematic reviews and meta-analyses of studies that evaluate healthcare interventions: explanation and elaboration. BMJ (Clinical Research ed). (2009) 339:b2700. doi: 10.1136/bmj.b2700, PMID: 19622552 PMC2714672

[18] Wei X Guo K Shang X Wang S Yang C Li J. Effects of different interventions on smoking cessation in chronic obstructive pulmonary disease patients: a systematic review and network meta-analysis. Int J Nurs Stud. (2022) 136:104362. doi: 10.1016/j.ijnurstu.2022.104362, PMID: 36206617

[19] van Hoogstraten LMC Vrieling A van der Heijden AG Kogevinas M Richters A Kiemeney LA. Global trends in the epidemiology of bladder cancer: challenges for public health and clinical practice. Nat Rev Clin Oncol. (2023) 20:287–304. doi: 10.1038/s41571-023-00744-3, PMID: 36914746

[20] Cirillo L Innocenti S Becherucci F. Global epidemiology of kidney cancer. Nephrology, dialysis, transplantation: official publication of the European Dialysis and Transplant Association-European Renal Association. (2024) 39:920–8. doi: 10.1093/ndt/gfae036, PMID: 38341277

[21] Yong C Brown JA Takacs EB. Performing medical education research in urology: challenges and opportunities. Curr Urol Rep. (2020) 21:45. doi: 10.1007/s11934-020-00997-w, PMID: 32889609

[22] Feng H Wang Y. Physiology education and teaching in Chinese mainland medical schools: the status quo and the changes over the past two decades. Adv Physiol Educ. (2023) 47:699–708. doi: 10.1152/advan.00020.2023, PMID: 37498549

[23] Liu X Feng J Liu C Chu R Lv M Zhong N. Medical education Systems in China: development, status, and evaluation. Academic medicine: Journal of the Association of American Medical Colleges. (2023) 98:43–9. doi: 10.1097/ACM.0000000000004919, PMID: 35947483

[24] Abrams P Brausi M Buntrock S Ebert T Hashim H Tiselius HG. The future of urology. Eur Urol. (2012) 61:534–40. doi: 10.1016/j.eururo.2011.11.005, PMID: 22137602

[25] de Vries AH Schout BM van Merriënboer JJ Pelger RC Koldewijn EL Muijtjens AM. High educational impact of a national simulation-based urological curriculum including technical and non-technical skills. Surg Endosc. (2017) 31:928–36. doi: 10.1007/s00464-016-5060-1, PMID: 27387182

[26] Ma X Zeng D Wang J Xu K Li L. Effectiveness of bridge-in, objective, pre-assessment, participatory learning, post-assessment, and summary teaching strategy in Chinese medical education: a systematic review and meta-analysis. Front Med. (2022) 9:975229. doi: 10.3389/fmed.2022.975229, PMID: 36186766 PMC9521335

[27] Liu CX Ouyang WW Wang XW Chen D Jiang ZL. Comparing hybrid problem-based and lecture learning (PBL + LBL) with LBL pedagogy on clinical curriculum learning for medical students in China: a meta-analysis of randomized controlled trials. Medicine. (2020) 99:e19687. doi: 10.1097/MD.0000000000019687, PMID: 32311943 PMC7220526

[28] Wen H Xu W Chen F Jiang X Zhang R Zeng J. Application of the BOPPPS-CBL model in electrocardiogram teaching for nursing students: a randomized comparison. BMC Med Educ. (2023) 23:987. doi: 10.1186/s12909-023-04983-x, PMID: 38129836 PMC10740289

[29] Hu K Ma RJ Ma C Zheng QK Sun ZG. Comparison of the BOPPPS model and traditional instructional approaches in thoracic surgery education. BMC Med Educ. (2022) 22:447. doi: 10.1186/s12909-022-03526-0, PMID: 35681190 PMC9185859

[30] Li Z Cai X Zhou K Qin J Zhang J Yang Q. Effects of BOPPPS combined with TBL in surgical nursing for nursing undergraduates: a mixed-method study. BMC Nurs. (2023) 22:133. doi: 10.1186/s12912-023-01281-1, PMID: 37088853 PMC10122814

[31] Cacciamani GE Okhunov Z Meneses AD Rodriguez-Socarras ME Rivas JG Porpiglia F. Impact of three-dimensional printing in urology: state of the art and future perspectives. A systematic review by ESUT-YAUWP group. Eur Urol. (2019) 76:209–21. doi: 10.1016/j.eururo.2019.04.044, PMID: 31109814

[32] Chen MY Skewes J Desselle M Wong C Woodruff MA Dasgupta P. Current applications of three-dimensional printing in urology. BJU Int. (2020) 125:17–27. doi: 10.1111/bju.14928, PMID: 31622020

[33] Youssef RF Spradling K Yoon R Dolan B Chamberlin J Okhunov Z. Applications of three-dimensional printing technology in urological practice. BJU Int. (2015) 116:697–702. doi: 10.1111/bju.13183, PMID: 26010346

[34] Frankiewicz M Vetterlein MW Matuszewski M. VR, reconstructive urology and the future of surgery education. Nat Rev Urol. (2023) 20:325–6. doi: 10.1038/s41585-022-00722-x, PMID: 36604520 PMC9813879

[35] Zattoni F Carletti F Randazzo G Tuminello A Betto G Novara G. Potential applications of new headsets for virtual and augmented reality in urology. Eur Urol Focus. (2023) 10:594–8. doi: 10.1016/j.euf.2023.12.003, PMID: 38160172

[36] Junbo B Jun M Hengqing A Wanfu L Jia L. Research on the application of the PBL-based BOPPPS teaching model in pediatric surgery teaching. Medical theory and practice. (2023) 36:3945–8.

[37] Lianhong D Yun Z Heng H. Application of BOPPS teaching mode in orthopedics theory teaching. China modern medicine. (2021) 28:186–9.

[38] Jia G Shaogan J. The application of BOPPPS combined with O2O to resident training of neurosurgery department. China Continuing Medical Education. (2020) 12:14–6.

[39] Kang H Qing Y Zhigang S. Research on the application of BOPPPS teaching mode in the clinical teaching of thoracic surgery. Health vocational education. (2022) 40:106–8.

[40] Zhuoqi J Weiru Z Jizhao W Zhe W Guangjian Z Junke F. Study on the application of BOPPPS teaching mode combined with 3D video-assisted thoracic surgery training in practice teaching of thoracic surgery. China medical education technology. (2023) 37:196–201.

[41] Jia L Wei Z Biliang C Hong Y Shujuan L. Application and evaluation of BOPPPS combined with CBL teaching methods in the clinical practice of gynecology and obstetrics. China Medical Herald. (2018) 15:68–71.

[42] Wei T Wei W. Application of BOPPPS and PBL in surgery teaching. China Continuing Medical Education. (2020) 12:39–41.

[43] Dawei W Ce S Huayang P. Application of BOPPPS teaching mode of online platform in general surgery practice. Continuing Medical Education. (2023) 37:53–6.

[44] Wei Z Yun T Liping Y Chuchu W Yanling L Jiejie Z. Application of mind mapping based combined BOPPPS teaching model in neurosurgery clinical nursing teaching. Journal of Chengde Medical University. (2023) 40:75–9.

